# Combination of Multi-Agent Systems and Wireless Sensor Networks for the Monitoring of Cattle

**DOI:** 10.3390/s18010108

**Published:** 2018-01-02

**Authors:** Alberto L. Barriuso, Gabriel Villarrubia González, Juan F. De Paz, Álvaro Lozano, Javier Bajo

**Affiliations:** 1 Faculty of Science, University of Salamanca, Plaza de la Merced s/n, 37002 Salamanca, Spain; gvg@usal.es (G.V.G.); fcofds@usal.es (J.F.D.P.); loza@usal.es (Á.L.); 2 Department of Artificial Intelligence, Polytechnic University of Madrid, Campus Montegancedo s/n, Boadilla del Monte, 28660 Madrid, Spain; jbajo@fi.upm.es

**Keywords:** birth sensor, bovine embedded hardware, ambient intelligence, virtual organizations of agents

## Abstract

Precision breeding techniques have been widely used to optimize expenses and increase livestock yields. Notwithstanding, the joint use of heterogeneous sensors and artificial intelligence techniques for the simultaneous analysis or detection of different problems that cattle may present has not been addressed. This study arises from the necessity to obtain a technological tool that faces this state of the art limitation. As novelty, this work presents a multi-agent architecture based on virtual organizations which allows to deploy a new embedded agent model in computationally limited autonomous sensors, making use of the Platform for Automatic coNstruction of orGanizations of intElligent Agents (PANGEA). To validate the proposed platform, different studies have been performed, where parameters specific to each animal are studied, such as physical activity, temperature, estrus cycle state and the moment in which the animal goes into labor. In addition, a set of applications that allow farmers to remotely monitor the livestock have been developed.

## 1. Introduction

The last decade saw a breakthrough in the field of Ambient Intelligence (AmI), resulting in an improvement in the quality of people’s lives. The main objective of AmI is to adapt technology to the needs of the people in a way that allows users to interact in a natural and effortless manner with the different systems that make up the environment. Technology must act in a transparent way by adapting to the individuals and their context; simplifying the accomplishment of daily tasks and the communication between them and the environment. To achieve this goal, it is necessary to develop new technological models that allow users to interact with multiple devices simultaneously. The devices must collaborate in the accomplishment of daily tasks without the individuals being aware of it. Due to the miniaturization of sensors and the reduction of costs in manufacturing processes, AmI can now provide new solutions to daily problems that were unthinkable just a few years ago. One field to which AmI can be applied to is that of livestock and natural resources management.

Currently in Spain, there are more than 23 million head of swine and more than 18 million of sheep, representing 15% of the total of the European Union [1]. The continuous growth that the farms are experiencing and the increasing demand for farm produce, make technology an essential instrument of continuous improvement in the development of farms. Requirements are also becoming increasingly strict for both the breeders and the livestock, so the study of factors such as food, physical activity or the animal’s health is necessary. On large farms, it is difficult to devote time to observing the behavior of each animal. Technology must provide solutions that simplify farmers’ work, especially in repetitive and difficult tasks, such as those that entail the study of the factors mentioned above. The research and development of systems that detect anomalies in animals at early stages, is vitally important. Numerous research groups are working on the use of electronic systems in livestock with the aim of improving productivity and reducing operating costs. AmI allows farmers to remotely access up to date information on the animals and obtain their complete traceability. The joint use of information technology and electronic devices, allows to monitor and study parameters as well as the consumption of energy on the farm, the level of food in the feeders, the lighting, climatology, the state of animals’ health, their physical activity, etc. Smart-farming or precision farming consists in applying information and communication technologies to livestock and agriculture. Its main objective is to increase the efficiency and quality of production through rapid decision making in cases where some type of problem arises on the farm.

This work presents a low cost wireless network sensor (WSN) interconnected through a multi-agent architecture based on virtual organizations. This system offers remote farm monitoring and traceability service. The aim pursued in this work is to save the farmer from unnecessary movement from place to place and to increase the productivity of the farm, reduce costs and risky situations. A WSN allows to obtain information on the environment and act upon it. The infrastructure of WSNs is capable of distributing communications in dynamic environments, increasing mobility and efficiency, regardless of the location. It is important to inquire into mechanisms that allow constructing computing agent platforms and communication agents that are portable to light devices.

The presence of heterogeneous protocols makes the integration of sensors difficult, since it makes it necessary to incorporate new communication systems between the different elements. When necessary, the proposed system will permit the sending of alerts to the mobiles of users, who will be able to visualize problems of each animal in real time. 

By using sensors, it will be possible to detect situations such as the beginning of labor or the estrus cycle, rumination problems, excess body temperature, the distance travelled, events and information of interest to the farmer. The novelty of the system is the implementation of a dashboard which allows people with little technological knowledge to visualize the state of the farm, using a TV.

The article is structured as follows: Section 2 reviews the state of the art and related projects; Section 3 describes the proposed architecture; and outlines the case study that has been conducted to evaluate the system; and finally the results obtained and the conclusions drawn from the work are presented in Section 4. In addition, Appendix A has been included with the hardware design schemes used in this work.

## 2. Background

Due to the miniaturization of hardware, devices are gradually reaching out to all the levels of society [2]. This fact, combined with the lowering production costs of consumer electronics, has favored the rise of Ambient Intelligence (AmI) in different areas such as robotics [3], health [4], education [5] or the transport industry [6]. In short, we can see how the application of AmI techniques has spread to practically every field.

The field of agriculture and livestock is no stranger to the development of AmI projects. In recent years, the implementation of different technological solutions has been promoted in order to move towards intelligent agriculture. In the case of the European Union, such initiatives have been supported by the European Parliament, as evidenced by two reports, which are of great importance to The Alliance for Internet of Things Innovation (AIOTI) [7]: “Technological solutions for sustainable agriculture in the EU” [8] and “Enhancing innovation and economic development in future European farm management” [9].

Smart Farming is based on the use of information and communication technologies, combined with tools that support decision-making and improve the efficiency, productivity and profitability of companies in this sector [10]. The use of intelligent devices allows improving traditionally used tools, by providing them with context awareness and intelligence, as well as automating the accomplishment of different tasks or allowing them to be performed remotely [11]. To create a Smart Farm, a network infrastructure is required which allows to connect different low-cost sensors and wireless actuators, used in the monitoring and remote control of facilities and livestock. Data collected by sensors (such as odometry of agricultural machinery, soil moisture, temperature, soil conductivity, pH level, animal position, body temperature, etc.) are transmitted to a data storage station, where they can be observed and analyzed using artificial intelligence techniques. Thus, farmers can increase their agricultural production through the statistics, patterns or predictions obtained from these data. Moreover, the use of actuators favors the automation and remote operation of certain tasks, such as cattle feeding, the use of irrigation systems, or temperature control in closed facilities where the livestock is located [12]. 

Santana-Mancilla et al. outline in their work [13] a set of practical considerations that should be taken into account when using and designing Wireless Sensor Networks (WSNs) for livestock farms. Some of the challenges tackled that work are related to: *(i) mobility*: the sensors used in the monitoring of animal parameters are subject to continuous changes of position, so the topology of networks and the routing of paths must be adapted and respond to the movement of animals; *(ii) interferences produced by the animals:* in many cases, animals are in herds and their own bodies, especially in cattle, can cause a significant reduction in the radio signal; *(iii) limitations in data storage:* due to the nature of low cost sensors, their capacity for data storage is limited, so it must be compensated with the use of robust protocols that allow to transmit the information collected by the sensors to central nodes; and *(iv) battery life:* since replacing batteries in livestock is an expensive process, the optimization of battery life in sensors is crucial; thus, we must work on reducing the energy consumed by data transmission.

Within the scope of livestock, AmI techniques have been most popularly implemented in the beef cattle industry. Given the complexity of breeding beef, the use of AmI allows farmers to perform certain tasks or detect possible problems that their animals may present. In the state of the art work, some of the most relevant studies apply AmI to the following fields:

- *Monitoring the position of the animal and analysis of the behavior of the herd*: By monitoring the position of livestock, it is possible to study the behavior of both individual animals and the herd as a whole. Since it may be argued that wearable technology annoys animals and alters their habitual behavior, studies that deny this theory have been carried out [14]. Wearable technology designed for animals usually takes the form of necklaces that make use of GPS, although there are also devices that make use of other technologies such as ZigBee [15]. Studies in which the position of cattle is monitored, vary in their objectives, some for example focus on assessing the animals’ periods of rest, ingestion and activity [16,17] and others on controlling grazing time in certain areas of the field [15]. In addition, the use of technology allows quickly locating cattle on extensive farms, which is normally a difficult task.
- *Intake control/feeding monitoring*: Ingestion, rumination and rest are the main activities of ruminant cattle. Thanks to their monitoring, breeders are able to take better decisions in relation to pasture management, as well as the diet supplements that the livestock may require [18]. There are several state of the art works that follow this line of research. For example, in [19], a system is proposed for the monitoring of feed intake in cattle; it is based on the combined use of scales located in self-locking feeders and proximity sensors worn by each animal. With this system, it is possible to identify the amount of food ingested by each animal. Although this is an effective system that accurately measures the ingested quantities, it is only suitable for intensive livestock farming. Other types of systems are required to measure feed intake on extensive farms. In the current state of the art works, the most common trend is the use of microphones to detect acoustic signals and classify them into different events, usually chewing, chewing-biting and biting. The works of Chelotti et al. [20], Clapham et al. [21] and Tani [22] are some of the works that follow this line of research.
- *Estrus detection*: One of the most influential factors in the productivity of livestock farms is the reproduction of livestock. In recent years, artificial insemination has become more frequent, since it has a higher reproductive efficiency [23]. Therefore, it is crucial to detect the moments in which the animals’ fertility levels are high. It is estimated that the duration of estrus is between 11 and 21 h; at the end of this period, ovulation occurs, which normally lasts 12 h. It is generally recommended to carry out artificial insemination for 6 to 24 h before ovulation [24]. Studies that apply AmI to detect estrus are mainly focused on: (i) *detection of heat events*—period of time before ovulation in which cows are sexually receptive; for example, in the work of Shahriar et al. [25], heat events were identified through the use of accelerometers and unsupervised learning; or (ii) *the monitoring of body temperature*—there are several studies which determine a relationship between the body temperature of cattle and estrus, in particular, with a mean increase of 0.9 °C in the vaginal temperature during estrus [26]. Numerous studies have been carried out in this regard, all of them employ sensors to measure the cows’ vaginal temperature; in some cases intravaginal sensors are used [27], while in others less intrusive sensors are used, such as in the work of Miura et al. [28], where vaginal temperature is measured by a sensor placed in the outer zone, at the base of the tail.
- *Calving detection*: Cows frequently experience calving difficulties; to prevent calf loss, human intervention is necessary in many cases. Thanks to calving prediction, cattle breeders are able to assist the parturient, this increases the chances of healthy calves being born and assures cows recover rapidly after calving (of vital importance in animals dedicated to milk production) [29]. In this regard, it is not only important to determine the time of delivery, but also to know the location of the parturient—especially on extensive farms. In some cases, sutured sensors are placed in the parturient vulva [30] to detect labor and a variety of other sensors is used to measure a number of factors; such as body temperature or parturient activity level [31]. The use of collars with integrated GPS systems [32] is the most widespread technique in the detection of animal position.
- *Syndromic monitoring systems*: Syndromic monitoring systems measure non-specific data that may be related to animal health [33]. The data collected by different sensors help to identify specific pathologies that are related to the appearance of diseases in cattle. In many lines of research, this type of systems is used. There are several studies in the literature, such as the work developed by Park et al. [34], where a complete set of sensors is used to create an animal health monitoring system, or in the work of Frese et al. [35], where electrodes are used to detect symptoms of gastrointestinal diseases related to arrhythmias.

Table 1 shows a collection of different wearables for cattle that can be found in the market today.

As stated above, different ambient and artificial intelligence techniques have been applied in this field of study. Thanks to the great advance of technology, a new point of view arises in the investigation of artificial intelligence [43]: Distributed Artificial Intelligence. It is dedicated to the study and definition of semi-autonomous computer systems that attempt to solve problems in which a collective behavior is more efficient than an individual one [44]. Within this area, we can talk about multi-agent systems (MAS), which deal with the intelligent coordination of a set of autonomous agents, including their knowledge, goals, properties and plans for solving problems or making decisions [43]. MAS have been successfully used in different domains thanks to their capabilities of solving computationally difficult (or spatially distributed) problems and their aptitude to develop social behaviors [45]. Currently, the idea of modeling systems through the iterations that occur between their agents is opening the way to a new trend: modeling the behavior of the system from an organizational point of view. There are different platforms that facilitate the design and implementation of MAS (e.g., Java Agent Development Framework (JADE) [46] and Mason [47]), but few that support virtual organizations. PANGEA [48], created by the BISITE research group, is an alternative which does support virtual organizations and offers inter-compatibility with multi-agent platforms using the FIPA-Agent Communication Language (FIPA-ACL). PANGEA employs methods that allow to reorganize and balance the load according to the computational needs of the system, using organizational concepts. It allows for case study modeling to be done using debugging tools, with a set of libraries for all types of IoT controllers and devices. Moreover, it offers a series of tools that facilitate platform creation and management in general. PANGEA provides the necessary elements to allow communication between agents in an easy and safe way. In this way, it favors the distribution of resources and facilitates the control of the nodes where the agents are deployed.

After a detailed review of the state of the art, we found that there was a research gap in a multi-agent architecture that would combine a variety of final services with the aim of improving livestock farm management. This is because all the works published so far have focused on detecting or resolving specific problems. The main objective of this work is to design a platform which makes use of technology that allows for the intelligent and remote management of a livestock farm. It was necessary to implement a MAS in the designed architecture due to the use of sensors with computationally limited hardware as well as the need for together with the need for the efficient use of the battery. These needs can be satisfied by a MAS which has lightweight and fast connection protocols. One of the benefits of multi-agent systems is their scalability, since they are inherently modular. This feature makes it possible to incorporate new features and capabilities much more easily than in a traditional system [49]. The use of a multi-agent architecture allows for the integration of any sensor, independently of its manufacturer; this makes it much simpler to conduct case studies. The multi-agent system PANGEA was chosen for this case study in particular due to its communication protocols, which can be adapted to the design and deployment of agents embedded in ATMega microcontrollers. The main novelties of this work are: (a) the deployment of computationally limited sensors using PANGEA; (b) the ability of the platform to predict estrus, calving, as well as the monitoring of cows’ feed intake; and (c) the design of a platform that allows monitoring and controlling a livestock farm remotely using low cost hardware as well as software that can be used easily by people who have limited knowledge of IT.

## 3. Proposal

This section details the architecture designed for the remote monitoring of a beef cattle farm. The following monitoring system detects heat and calving time and measures the levels of feed in the feeders. In addition, several applications are developed which allow to monitor and visualize the farm remotely, from a TV, computer or mobile phone.

The proposal is organized into several subsections to reduce its complexity. Section 3.1 addresses the topic of the MAS platform design and outlines the different virtual organizations that constitute the proposal. Section 3.2 deals with the deployed data connectivity. Section 3.3 describes the sensors used. Section 3.4 describes the employed sensing techniques. Finally, Section 3.5 outlines the applications developed for end users.

### 3.1. Multi-Agent Architecture

To conduct the case study, we decided to develop a MAS based on virtual organizations. The use of a MAS that allows to develop virtual organizations of agents (VO) provides the system with flexibility and dynamism; it equips the platform with new adaptability, reorganization and learning capabilities. The agents that orchestrate the system belong to different organizations, depending on the nature of the functionality that they perform. VO group agents with similar roles in one organization, with the aim of retrieving, merging and processing the information in an automatic way. This makes the proposed system extensible, favoring a high adaptability to the real demands of the environment. Since the proposal is oriented towards the modeling of MAS as a virtual organization of agents, we must be able to define structure, roles and hierarchies, as well as a series of rules that regulate interaction and communication between agents. PANGEA is perfectly suited to the needs of the environment in which we work, where it will use agents embedded in different wireless sensors. PANGEA also allows for the quick and simple deployment of agents embedded in computationally limited devices.

**Figure 1:**
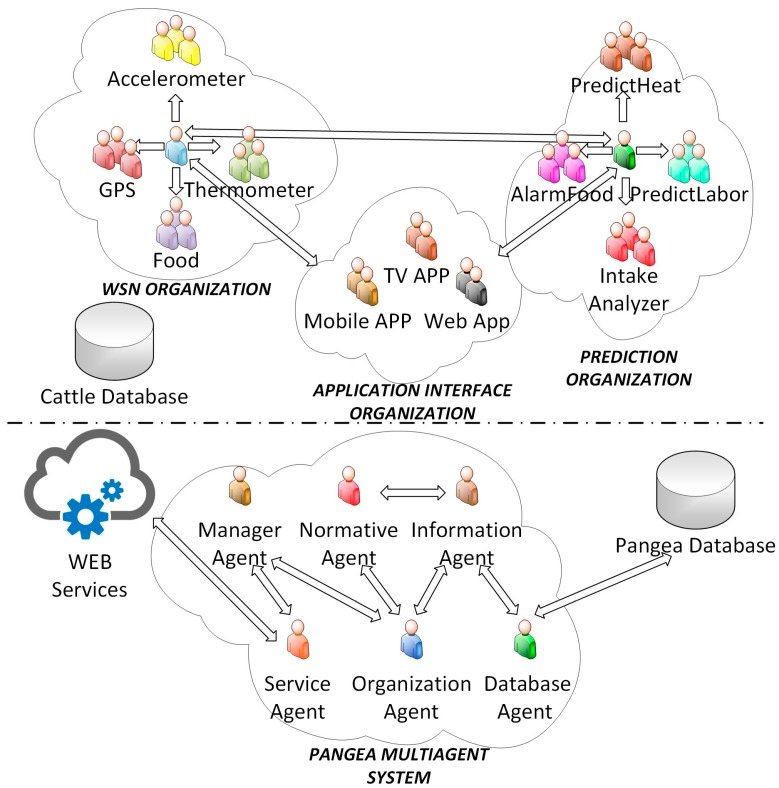
Virtual organization of agents overview.

As can be seen in Figure 1, the general architecture of the system is made up of two large blocks: the lower part shows the agents corresponding to the PANGEA platform, while the upper part shows the virtual organizations of the agents in the proposed system, which are: *WSN Organization, Prediction Organization* and *Application Interface Organization*. The main tasks of each of the organizations within the system are listed below:

- *WSN Organization*: It is responsible for adapting the information of the different hardware sensors that are used in the case study, to a protocol or common language that can be read by all the elements of the platform. The main function is to adapt or translate the raw data into a JSON-type format. This organization is made up of *Accelerometer, GPS, Thermometer* and *Food agents*. Each of these agents has the functionality that it needs to be able to obtain the information of each of the sensors. Each of the agents mentioned above, communicate only with a central agent who is responsible for the organization. The main capability of this agent is to establish a bidirectional communication with entities that do not belong to the same virtual organization. These entities may consist of agents that access databases, or other virtual organizations of agents that interpret or analyze the data.
- *Prediction Organization*: It is responsible for retrieving the data obtained by the WSN organization and processing them. The objective of each agent will be to detect or anticipate specific events that are crucial for the farm’s productive processes of the cattle yield and to send an alarm. The agents within this organization analyze events associated with animal feeding, calving or estrus (*AlarmFood, predictHeat, and predictLabor* agents). When an event is detected by any of these agents, it is communicated to the central agent who is in charge of notifying this event to the rest of the agents in other organizations.
- *Application Interface Organization*: For the end user to be able to view the information that has been collected and analyzed, it has to be represented by a software application. The central agent of this organization is responsible for obtaining the heterogeneous information from other organizations to adapt it to the standards used in the different applications, where the agents responsible for collecting this information are placed for later viewing: *Mobile App, Web App,* and *TV App* are the agents responsible for the visualization layer of the applications for mobile, web and Smart TV respectively.

Furthermore, the proposed system makes use of a set of agents that are part of the PANGEA MAS [48], which has been used as a base line for this work. These agents are described below.

- *Manager agent*: It is responsible for supervising tasks, ensuring that the system works correctly, checking that there are no overloads in the different functionalities of the system or that errors of communication between agents do not occur.
- *Information agent*: When a new agent becomes part of the organization, the agent notifies the *information agent* of the services it offers. In this way, the *information agent* is aware of all the services available in the organization, thus being able to serve as a directory which the other agents can consult to know of the services that they can access.
- *Service agent*: This agent is responsible for recording and controlling the operation of the services offered by agents. In addition, it works as a gateway, as it is in charge of the distribution of the services that offered by the agents through web services, so that they are accessible from outside the organization.
- *Normative agent*: This agent ensures compliance with all rules defined in the organization, always ensuring that agents use those services for which they have permission, warranting the security of communications made within the system.
- *Database agent*: This agent in charge of accessing the PANGEA database to obtain the pertinent information. This database agent is responsible for representing and storing the contextual information of the system: what organizations and sub-organizations of agents it contains, what type of topology they form, what roles the different agents fulfill, what services they offer, etc.
- *Organization agent*: This is the agent in charge of managing organizations and sub-organizations. It is responsible for verifying the entry and exit of the agents to the system, as well as assigning a role to each of the agents in the organization.

One of the greatest advantages of the use of PANGEA is that it allows for the use of different communication protocols, such as HTTP or Message Queue Telemetry Transport (MQTT) among others. MQTT is a messaging protocol specially designed for communication between embedded devices with few computational resources, with low bandwidth and in high latency networks. Due to these characteristics, this protocol has been widely used in the field of Internet of Things (IoT). To verify that the use of this type of protocols provides greater advantages when used in machine-to-machine communication, than the advantages it offers when used with traditional systems based on HTTP protocols with service-oriented architectures, a comparison has been made between both paradigms.

**Figure 2:**
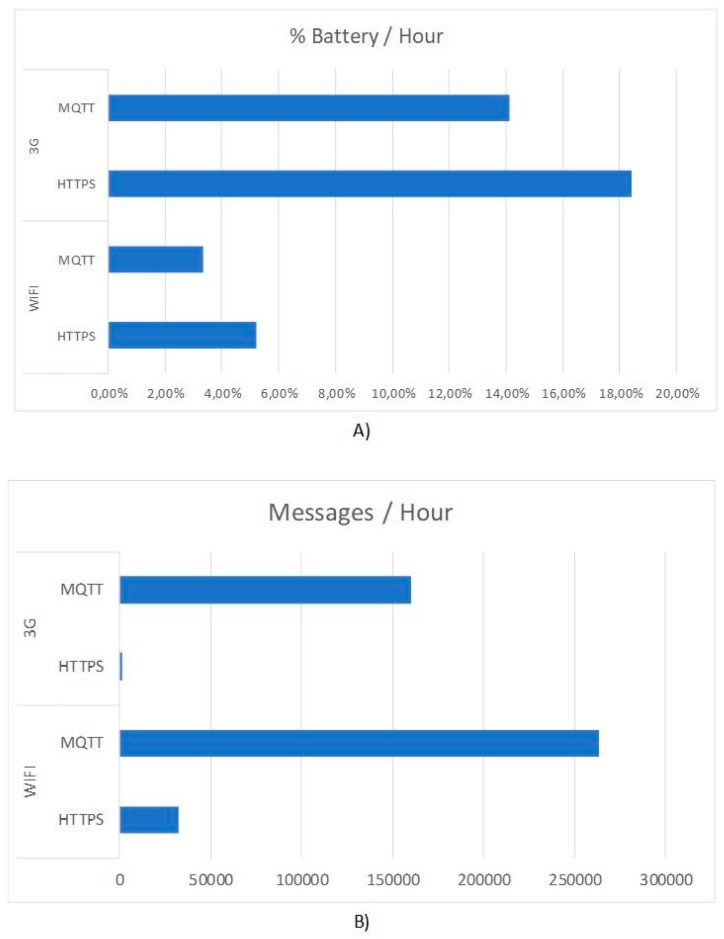
(**A**) Battery percentage consumed per hour and (**B**) messages sent per hour (results grouped by protocols and networking technologies).

This comparison is shown in Figure 2, the illustrated results have been obtained after sending identical messages from an agent embedded in the feeder devices under the following conditions: MQTT over 3G, MQTT over WiFi, HTTP over 3G, and HTTP over Wi-Fi. Figure 2A shows the percentage of battery consumed by the device during one hour and Figure 2B shows the number of messages sent in one hour for the four conditions indicated above. In either case, it can be seen that the choice of MQTT vs. HTTP is properly justified, not only for efficiency in sending messages (which is outstanding in the case of 3G networks) but mainly for the battery saving rates it offers. This aspect is very important in a system like the one proposed in this work, since the replacement of batteries is an expensive process for the cattle ranchers, so the battery saving in the different sensors, also supposes a greater efficiency in productive processes. The flexibility offered by PANGEA enables the use of the most suitable protocol for each use case where, in the case of sensors communication, MQTT has been proven being appropriate.

**Figure 3:**
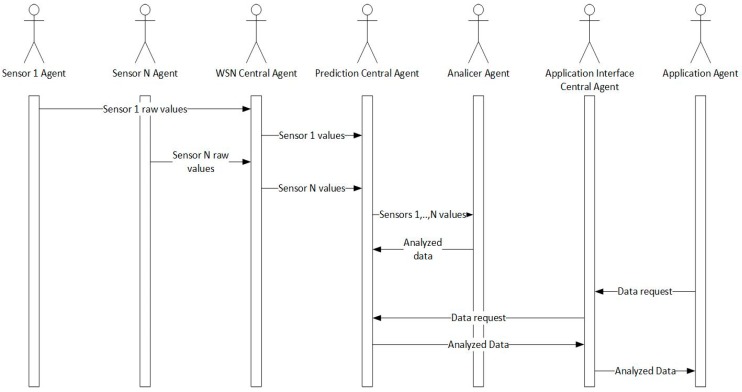
Interaction performed between agents.

The general operation of the processes occurring in the platform can be seen in Figure 3. In the first place, the agents embedded in the sensors carry out a data acquisition process. These agents send the collected raw information to the WSN Central agent. This agent is in charge of storing the received information in a standardized way, as well as sharing this information with the other organizations, restricting their access only to the services that require it. To do a data analysis or prediction (such as labor or heat detection), the WSN Central Agent will be responsible for providing information from the different sources to the Prediction Central Agent, which will in turn send it to the corresponding Analyzer Agent. These agents present reactive behavior defined by rules, and are responsible for performing the data analysis. After establishing this information, the analyzer agent will send it to the Prediction Central Agent, who is responsible for storing it so that, if an agent needs it, it is sent on demand (or in other cases, information can be spread from this agent to others automatically, as in the case of estrus detection). When a user makes use of a certain application (mobile application, web application or Smart TV application) and wants to view some kind of information, the software agent which is part of the application will make a request for the desired information to the Prediction Central Agent, which will be returned if available. The communications are controlled (although it is not represented in the figure in order to make it simpler to understand) through the PANGEA layer, which already includes rules that define communications between agents. The Normative Agent ensures that agents in the platform only use those services for which they have permission.

### 3.2. Farm Connectivity

**Figure 4:**
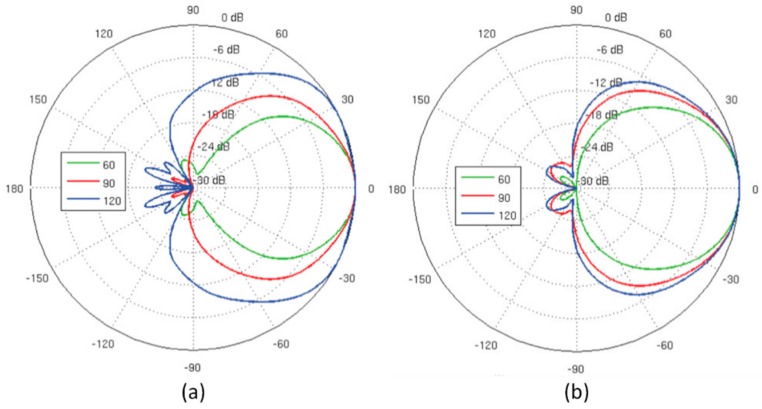
Behavior of the used antennas signal: Ti AM-V5 G-Ti (5 GHz): (**a**) horizontal Azimuth and (**b**) vertical Azimuth.

To provide a tele-monitoring service on the farm, data connectivity is required. To deploy a data network and offer an internet connection on the farm, several possibilities were studied. One of the alternatives was the hiring of a satellite line based on WiMAX technology. The necessary equipment had a high cost and the monthly quota of the data line was exorbitant, reason why this option had to be discarded. An alternative idea was to hire a conventional ADSL line. However, no service provider had coverage in the area where the farm is situated (40.796575, −6.241120). Finally, we thought of making a radio link. The farm where the connection was to be established is located near a town called La Fuente de San Esteban, Salamanca (40.8013959, −6.2624964). This core had a pre-installed broadband connection in the City Hall. To be able to share a data connection in a legal and free way with the inhabitants and farmers of the area, a collaboration agreement was signed with the Regional Government of Salamanca, Junta of Castile and León. This agreement required that, to share the Town Hall connection with the rest of the inhabitants and workers in the area, each connection should be limited to a 512 Kbit/s downstream speed. The farm did not have direct view with the Town Hall that it needed to connect, since it is located in the center of the village and is surrounded by houses, thus an intermediate point was chosen to act as a gateway. The highest point of the municipality was the Silo (40.795884, −6.251096) with a height of 50 m above ground level, and had electrical connection. In the upper part of the roof of the Silo and in each geographical orientation N, S, E, W an Ubiquiti brand antenna of type Ti AM-V5 G-Ti (5 GHz), was installed with a sectorial opening configuration of 120 degrees and a resulting gain of 15 dBi. Figure 4 shows the behavior of the signal in the antennas used with an aperture of 60°, 90° and 120° [50].

**Figure 5:**
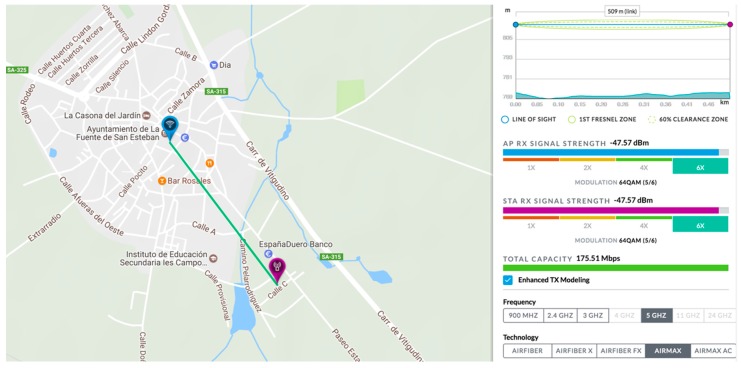
Altimetry and quality of the radio link.

Once the place where the connection could be made was determined, different point-to-point connections were made with nearby urban nucleus that did not have any kind of broadband connection. The radio link between the silo and the Town Hall was made using a 5 GHz equipment (PowerStation5 antennas [51]). Figure 5 shows the altimetry profile of the ground between the two points, where it can be verified that there is direct vision and that the quality of the link is correct, with a capacity of 175.51 Mbps.

**Figure 6:**
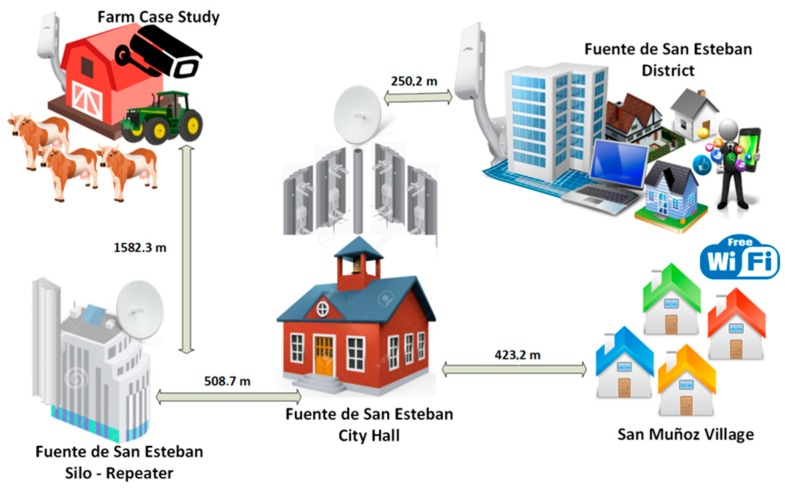
Network architecture diagram.

The modulation of the antennas in the link is 64 QAM using a polling policy based on Time Division Multiple Access (AIRMAX) that prioritizes the most active systems through a technique of intelligent surveys, achieving a greater use of the medium. Figure 6 illustrates the network architecture, the distances between each place and actual photos of the antennas.

From the image, we can see that the data connection that already existed in the municipality is distributed and is redirected to the highest point in the town to be able to share that connection with another urban nucleus that did not have a basic Internet connection. Nodes that want to connect to the Internet, such as laptops or smartphones that are approximately 300 m away from the town hall or silo do not require additional hardware to use the connection. Since the case study contemplates the possibility of monitoring the field by video streaming, we decided to use a NanoStation LOCO M5 5 GHz from the Ubiquiti brand, configured in client mode with a throughput of 150 Mbps.

Thanks to the network infrastructure described above, it was possible to deploy the proposed platform on the farm and to conduct the case study that proves the system’s feasibility.

### 3.3. Sensors in Livestock Farming

**Figure 7:**
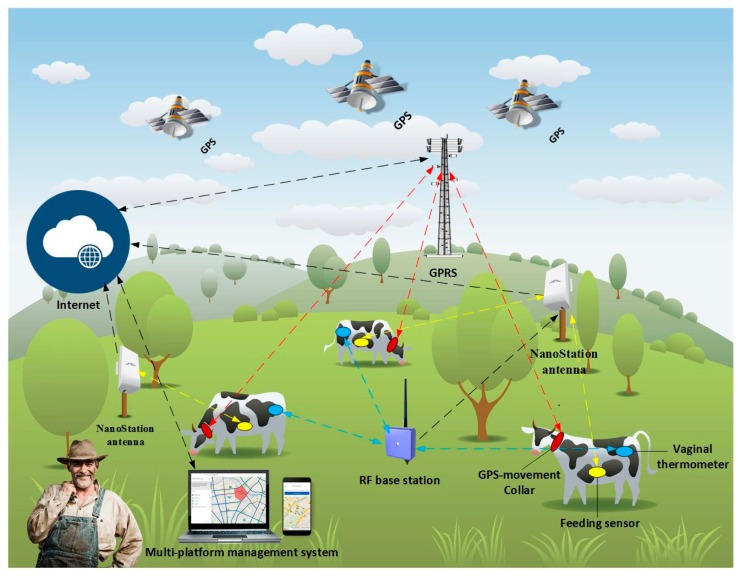
Infrastructure of the proposed system.

Figure 7 shows the general structure of the proposed architecture in the case study. The central part of this system is a WSN, whose main objective is the collection of different data from sensors placed on the cattle that are part of the farm. Depending on the type of sensor, the collected data will be sent in different ways: radio frequency, GPRS or Wi-Fi. To support these types of communication, it was necessary to incorporate additional communication elements, such as radio frequency stations or the previously mentioned NanoStation LOCO antennas. In either case, the final destination of the data is the same: a central server in which these data are stored for later analysis and interpretation. With artificial intelligence techniques, it is possible to automatically detect different events, whose identification can be of great help to the farmer. Thanks to the use of different applications, the farmer can receive alerts which notify when these events occurred. Additionally, he is also able to check the data collected by the sensors in real time. The following sections detail the operation of each of the sensors and the analysis performed on the collected data.

**Figure 8:**
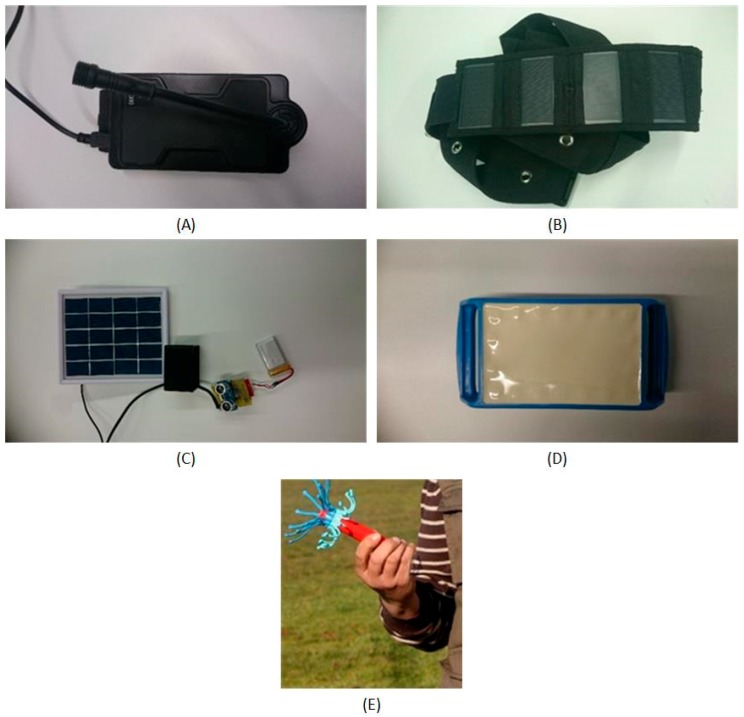
(**A**) GPS sensor; (**B**) solar collar for GPS sensor; (**C**) food sensor (ultrasonic sensor and solar battery); (**D**) motion sensor; and (**E**) vaginal thermometer.

In this organization we have GPS, movement, temperature and food sensors. The sensors that were used can be seen in Figure 8A–E.

The GPS sensor retrieves the GPS locations and the time stamps associated with each of them. This sensor has a SIM module that sends the location data to a server via GPRS. With the purpose of making this device energetically self-sufficient, a solar collar that charges its battery has been included. Additionally, a motion sensor is included, so the GPS is only activated when activity is recorded. This motion sensor is also used to detect the activity of the animal, since, as mentioned in the state of the art, a change in the activity of the animal is related to estrus.

As to the thermometer used, it is a vaginal thermometer that sends the data by radiofrequency to a base that subsequently sends them to a server, from which the information can later be extracted. The frequency of sending can be parameterizable, in this case study every 45 s. In the case the sensor cannot transmit the value to the RF base for some reason or because the distance between it and the sensor is too great, the sensor has an internal memory that temporarily stores the values, until the connection with the base RF is successfully reestablished.

The food sensor incorporates ultrasound sensors to measure the volume of feed. The sensor incorporates an Arduino-based controller to retrieve sensor information from the sensor and then send it remotely via WiFi technology to the remote server. In addition, the sensor includes a battery and a photovoltaic panel that makes the device autonomous; there is no need to connect it to electricity. To be more efficient energetically and thus have low consumption rates, sensor data are sent every 60 min. A low cost solar plate is used, with an operating voltage of 5 V, an output power of 1 W and a current of 0–200 MA.

As a summary, Table 2 shows which kind of data each sensor collects, and in which systems of analysis or prediction the data collected by the sensor are used. The detailed description of each of the analysis and prediction systems is shown in the next section.

### 3.4. Analysis and Prediction Systems

The agents are capable of predicting each of the problems posed in this case study. Some of the sensors require a system that is adaptive for each of the animals and that evolves with time. However, other sensors only require the definition of predefined rules, whose behavior as a result is reactive. In the next subsection, the functioning of sensors is described.

#### 3.4.1. System for the Detection of Feed Levels in the Feeders

**Figure 9:**
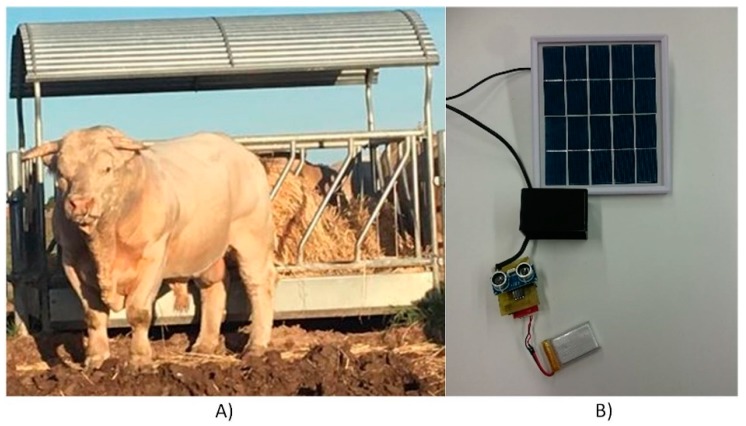
(**A**) Feeder; and (**B**) measuring system.

To implement this system, ultrasonic HC-SR04 sensors have been used. These sensors allow measuring distances in a range of 2–450 cm, with a 0.3 cm accuracy. To verify the correct functioning of the system, sensors have been installed on 4 feeders. Figure 9A shows one of the feeders where the sensors are installed. The sensors are placed at the base of the feeder’s roof. Since there are no electricity sockets close to the feeders, batteries have been chosen to supply power to the sensors. Moreover, photovoltaic plates have been used to charge the batteries and provide the system with complete autonomy. Figure 9B shows the sensor, battery and photovoltaic panel that were used in the case study.

To measure the level of feed, the sensor is installed at the base of the roof of the feeder and measures the distance to the base on which the food is placed. The distance from the roof to the base is 210 cm. On this farm, cattle are fed with bales of straw which measure 90 cm × 100 cm × 210 cm on average. A single bale is placed per feeder when feeding the cattle. It can therefore be extrapolated that a measurement of 210 cm recorded by the ultrasonic sensor corresponds to a level of feed of 0% (the height of the ceiling relative to the base), and that a measurement of 110 cm in the height between the ceiling and the bale), corresponds to a 100% feed level. In view of maximum and minimum values of feed in the feeders, the levels of feed can be calculated with the data collected by the sensor. There may be straw residues when new bales are being placed in the feeders, which will cause the feed height to exceed the upper limit that determines 100% of the feeder filling. In these cases, the level of feed will also be considered to be 100%.

#### 3.4.2. System for the Monitoring of Ingestion

A fundamental aspect of livestock farms is the correct feeding of livestock, since it has a direct impact on production rates, whether meat or milk. Although cattle feed on forages, the incorporation of concentrated foods in their diet helps to improve production to a great extent. For this improvement to be effective, it will be necessary to elaborate optimal feed portions for different groups of animals (steers, heifers, bulls, gestating cows, dry cows or production cows). This, in addition to boosting farm production, will in many cases help reduce costs, since the unnecessary use of feed will be largely avoided. To be able to correctly plan animal feeding, a system that allows for the monitoring of the animals’ feed intake, has been incorporated in the platform.

To control the animals’ feed intake, we developed a system which establishes what kind of food an animal has been ingesting (and for how long). Two sensors placed of the animals’ collars have been used to collect the necessary data. These sensors consist of an accelerometer and a GPS module, in which the agents are embedded: the *Accelerometer agent* and the *GPS Agent*, both belong to the *WSN Organization*. The main function of these agents is to collect the information from the sensors and transmit it to the central agent of the organization, which is responsible for providing the information from the sensors’ different heterogeneous sources to other organizations, such as the *Prediction Organization*. In this organization, the *Intake Analyzer* agent is in charge of merging information with the data recorded by the *Accelerometer agent* and the *GPS agent*. On the one hand, thanks to the data variation analysis done by the accelerometer, we can determine when the animal was eating or performing another activity. Furthermore, when geofencing techniques (to delimit the geographical areas where the feeders are located) are applied to GPS data, it is possible to determine if the animal is in an enclosed or open area. Thus, with the combined use of both sources of information, it can be specified whether an animal was eating or not, whether it was in one zone or another and thus, we can infer what kind of food it ingested; pasture in the intensive area or concentrated food in an enclosed area.

To develop a system that differentiates the animal’s intake periods from the data recorded by the accelerometer, tests have been carried out with data obtained from 6 Charolais breed cattle during a three-week period. During this period, these animals wore a collar that incorporates a LIS3DH accelerometer, which allows acceleration measurement in three axes, with a dynamically configurable detection range of ±2 g/±4 g/±8 g/±16, and which offers a base consumption rate of 6 μA in low-power mode. The procedure to establish if grazing activity is occurring, is the one described by Rayas-Amor et al. in their work [52]. This procedure establishes a relationship between the acceleration and tilt that can be used to estimate the grazing time. On the one hand, the median acceleration readings in the *x* axis in a range between 0.175 and 0.95 m/s^2^ establish grazing activity. On the other hand, the degree of vertical tilt in the y axis can be used to establish grazing position in a range of readings between 0° and 61°.

**Figure 10:**
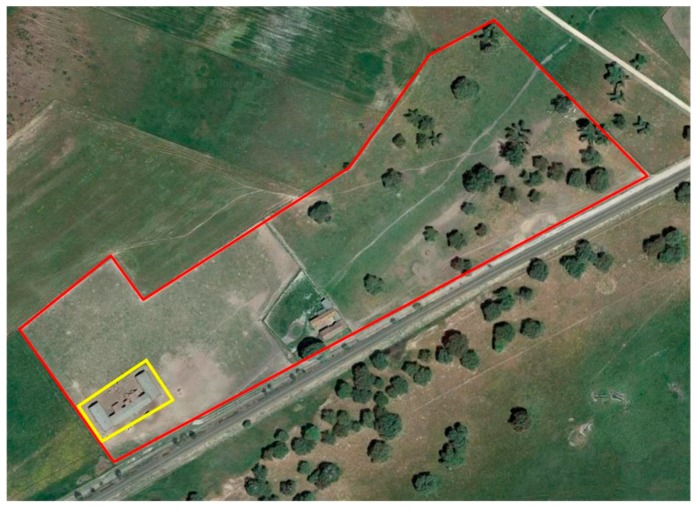
Aerial view of the farm, virtual enclosures.

The second component involved in the control of intake, aims to determine if the animal has been ingesting pasture or concentrated feed at a given time. There are two well differentiated areas within the farm where the study was carried out: the area where the feeders are located, and the extensive pasture area. As explained previously, it will be possible to determine whether an animal has been ingesting either grass or concentrated feed, based on the animal’s geographical location (by differentiating if it is in the zone of feeders or on extensive pasture). To develop this system, GPS sensors Neo-6 m, placed in the collars of the animals, have been used to continuously monitor the position of the animals. Thanks to the position provided by the GPS, and using geofencing techniques which allow to encircle a certain geographical area, it will be possible to determine if an animal is within one of these zones at any given time by means of a simple system of rules. Figure 10 shows the aerial view of the farm where the study was carried out. Two enclosures can be seen on the photograph, a yellow one and a red one, which correspond to the virtual fences. The yellow fence delimits the geographical space where the feeders are located and the red one delimits the geographical space of the whole farm. Thanks to the delimitation of these zones, we can establish if: (i) the animal is in the zone of the feeders; (ii) the animal is in the area of extensive pasture; or (iii) the animal is outside the farm area.

#### 3.4.3. System for Estrus Detection

The motion sensor is used to predict estrus. Every hour, the motion sensor sends the information from the accelerometers to the central server, where the activity of the animal is estimated. The procedure that is followed to calculate activity is the one described by Yin et al. [53]. Basically, the algorithm for a time interval of 5 s takes the mean acceleration values for the x-axis, *y*-axis, and *z*-axis and then calculates the value difference for the *x*, *y*, *z* axis between two contiguous intervals. These three values are used together with the sum of their absolute values in order to calculate the activity of the animals (Equation (1)).

(1) $$d_{i}=\left\{x,y,z,\left|x\right|+\left|y\right|+\left|z\right|\right\}$$

From the values, three clusters are created by applying PAM, so that groups g1, g2, and g3 have one for high, medium and low activity (Equation (2)). In the original algorithm, k-means was used, but to avoid problems with atypical values, PAM is used.

(2) $$G=\left\{\left\{g_{1},g_{2},g_{3}\right\}/g_{1}\cup g_{2}\cup g_{3}=\;\cup d_{i},g_{1}\cap g_{2}\cap g_{3}=\emptyset \right\}$$

To subsequently determine whether the cow is in heat or not, it is necessary to compare the number of elements in each group, with the elements from the groups obtained in previous days at the same hour. We used the indications provided in the work of Yin et al [53] to calculate the activity index of a cow for a particular time t.

(3) $$a_{t}=\overset{3}{\underset{i=1}{\sum }}\#g_{i}*w_{i}$$

where $\#g_{i}$ is the number of elements in group *i*, and $w_{i}$ is the weight of group *i*, by default in this order: {0, 0.1, 0.9}.

To calculate the activity index, compare the number of the value of Equation (3) according to the three days prior to the same hour as well as with the previous hour.

(4) $$v_{i}=\frac{3a_{t}-\left(a_{t-24}-a_{t-48}-a_{t-72}\right)}{\left(a_{t-24}-a_{t-48}-a_{t-72}\right)}+\frac{\left(a_{t}-a_{t-1}\right)}{a_{t-1}}$$

To consider aspects associated with the movement of livestock or situations in which cattle may be more active e.g., food, strangers on the premises, animals present, etc. The above formulas are modified to consider changes in the general activity of the control group of cows. These situations are established using Equation (5).

(5) $$I_{t}^{c}=v_{t}^{c}-\left|{\bar{v}}_{t}\right|$$

where $v_{t}^{c}$ is the activity index, calculated from Equation (4) for the cow *c* in the particular time *t*, $\left|{\bar{v}}_{t}\right|$ is the average activity index for all the cows in *t*. By applying Equation (5) it is possible to determine a variation in the activity index of the animals, in comparison to the cows that were previously used as a reference for this study. The selected animals for this study correspond to cows that are already pregnant and can be observed visually, or with cows which are already pregnant that are not observed visually, but the estrus has not been detected during three periods.

The estrus detection thresholds obtained by Equation (5) based on data are established manually at the beginning.

#### 3.4.4. System for Calving Detection

In cattle farming, human assistance is often required during labor to ensure a birth without any complications, safeguarding the health of both the parturient and the calf. If the farm is large or if the calving takes place at night, the danger is even greater; the farmer must look for the cow in the whole field and loses critical time on which the health of livestock depends. Thus, it is crucial for the farmer to know exactly the time and the geolocation of the place where the birth will start.

To solve this problem, a sensor has been designed consisting of an accelerometer and a temperature sensor, which is introduced into the cow’s matrix. This sensor should be placed when the following symptoms are detected in the animal: presence of colostrum in the mammary glands, decrease in body temperature or repetitive tail lifting.

**Figure 11:**
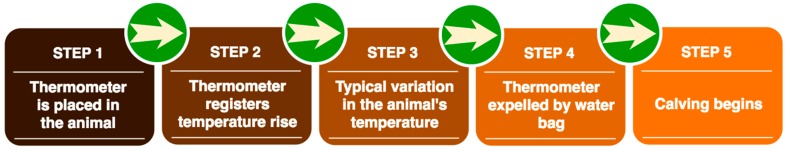
Steps in the detection of labor.

To detect labor, continuous monitoring of the animal’s temperature is performed. It is possible to detect calving in an early and approximate way when it is going to occur through the analysis of the cow’s body temperature. According to the studies conducted by Lammoglia et al. [54], the body temperature of the animal is significantly reduced in the 48 to 8 h prior to the labor. On the other hand, when the cow’s water breaks, the sensor is ejected out of the matrix, producing a variation in temperature, thus the exact moment at which labor begins, can be detected. The geolocation of the cow is obtained by using the GPS module that the cow carries on a collar and is described in detail in Section 3.4.2. The changes in temperature are determined empirically using the values collected by the temperature sensor. To implement this functionality, it is necessary to establish a temperature threshold that determines the beginning of labor. When the threshold is crossed, the farmer is alarmed that the cow is in labor. Figure 11 shows the steps that follow the procedure described above.

### 3.5. Developed Applications

It is very well known that the world population is getting older. The large number of elderly people in our society can be explained with improved health care and earlier retirement. Despite the internet boom, the older generation is still quite unfamiliar with the use of new technologies. Some are unaware of the benefits offered by technological devices while others, who are interested in using electronic applications and devices, find it too complex to manage them. The distance between technology and older people gets even greater when we look at its high cost. We must take into account that the majority of farm owners are elderly, and therefore the technological solutions offered for farms, should be economic and easy to use. The province of Salamanca has the largest number of cattle in Spain, a total of 6012, thus this work intends to provide a solution that will allow elderly farmers to correctly control and supervise their farms.

A monitoring prototype has been developed where farmers can view the data on their farm and their animals. They will be able to view these data from their own homes. We chose a low-cost platform design that allows to remotely interact with the farm. A human-machine interface (HMI) has been implemented so that people who have very little knowledge of technology can easily view data and receive notifications. An application for the television has been developed because elderly people are familiar with its use (unlike other electronic devices; computers, mobiles). Users manage the application with a remote control, whose colored buttons are associated with different actions or give access to the submenus of the application.

An alternative is offered to users who are familiar with new technologies; they can display all the data on a website. This website shows real-time information collected by sensors, including the location of animals, cameras, food sensors, water level sensors and alerts on heat, calving or feed levels.

Additionally, an Android application for mobile devices has been developed, where users can also view information and alerts on heat, calving and feeding. This application can be very useful to breeders, because when the system detects an important event, a notification is sent to the user’s mobile. This provides users with greater comfort and security since they do not have to be constantly checking the application in order to stay informed of the current state of the farm.

## 4. Results and Conclusions

The experimentation and validation phase has been carried out on the Ángel Santiago García Martín cattle farm, located in the town of Fuente de San Esteban town, in the province of Salamanca. The use of PANGEA as the MAS architecture permitted us to design a complex case study where computationally limited device participate in a reduced period. By using the designed architecture, the cattleman ubiquitously tests the algorithms implemented in the platform, allowing for their continuous improvement. The farm where the platform was implanted, has 450 hectares in total, out of which, 300 hectares are dedicated to bovine cattle. The number of livestock on the holding is 200. For the verification tests, a sample of 24 specimens belonging to the Charolais breed have been used.

For the development and validation of the intake monitoring system, tests were carried out with six Charolais breed cattle during a three-week period. Regarding the system that allows establishing what the periods of grazing have been, the use of tri-axial accelerometers has been previously probed to be accurate to estimate grazing time per cow and per day [52], results that could be empirically tested by observing the animals. Regarding the second component involved in the control of the intake, which aims to determine if the animal has been ingesting pasture at a given time, the accuracy of this system is subject to the accuracy of the location obtained by the GPS sensor. During the validation of the system, empirical tests were performed to check the possible fluctuations of the collected values, reaching 3 m accuracy in the measurements. However, the area around the location of the feeders in this place is so frequented by animals, the grass does not for 3–4 m around it. Thus, the error derived from the measurements in this case is minimal, since there is no food other than the one in the feeders in this space. However, the accuracy of this system in a farm where these conditions are not met, will be subject to the accuracy of the GPS in that location. To deal with this possible problem, a viable alternative may be the use proximity sensors, whose application has been validated in other works [19].

**Figure 12:**
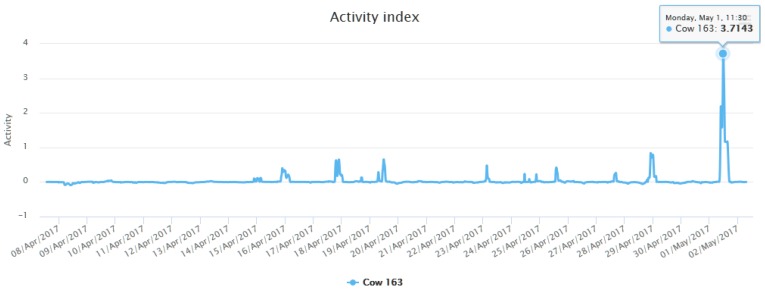
Activity index of a cow.

To study the relationship between an increase of physical activity of cows with the onset of heat, a motion sensor was placed in a sample of 8 adult cows. As discussed in Section 3.4.3, an algorithm is applied to calculate the cows’ activity index. Figure 12 shows the calculated activity index for one of the cows that participated in the study. During the study, a pronounced increase in the activity index was discovered, as can be seen in the Figure 12, it coincides with the beginning of the animal’s zeal. This system has been tested in previous works reaching a 100% success rates [53]. In this case study, the relationship between the increase in the activity index and the beginning of zeal has been validated by a human expert: the head farmer. The number of the detected heat events has matched with the observed heat events in eight out of the eight studied cows.

To study the temperature variability prior to calving, we opted to monitor six cows on the farm. The objective was to study the temperature pattern to be able to adjust the sensitivity of the designed sensor and to study the variation of the cows’ temperature over these days. Table 3 shows the data obtained for six days prior to delivery and the evolution of the temperature variable at the time of delivery. Three of the cows studied were pregnant with a male offspring, while the other three were females.

**Figure 13:**
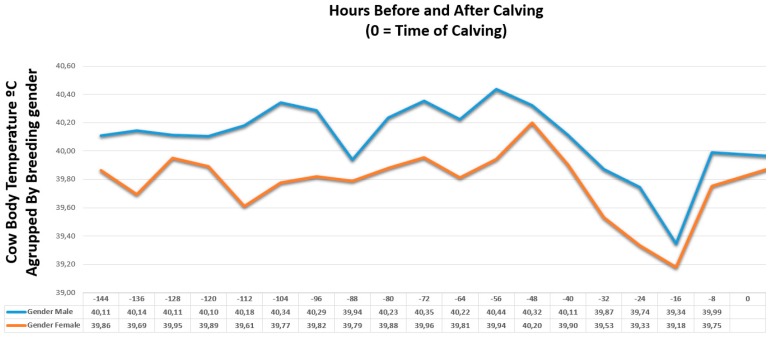
Cow average temperature, grouped by breeding gender.

Figure 13 shows the evolution of the average temperature of the offspring according to their gender. As it can be seen in the image, the temperature of the cows lowers 48 h before the calves were born and when the cow gives birth, the thermometer is expelled. Once it is expelled it measure the temperature of the environment, in this way it is easy to predict labor. In view of the obtained results, the system performance has been verified in all the cows of the control group. The accuracy of this method is subject to two factors: the correct functioning of the temperature sensor and the outside temperature. Assuming that the sensor works properly, the detection of delivery would only fail when the ambient temperature is exactly the same as the body temperature of the cow. Considering that the body temperature of these animals is around 40 °C, and that in these cattle farm the deliveries are scheduled for the months of November, January and February, there is no match possibility between both values.

One of the main results of this work is a monitoring platform that allows to check the state of the exploitation in real time, as well as to receive different alarms of the events detected by the system. As explained in previous sections, in this work, we opted to implement three different applications: one for TVs, one websites and one for smartphones.

**Figure 14:**
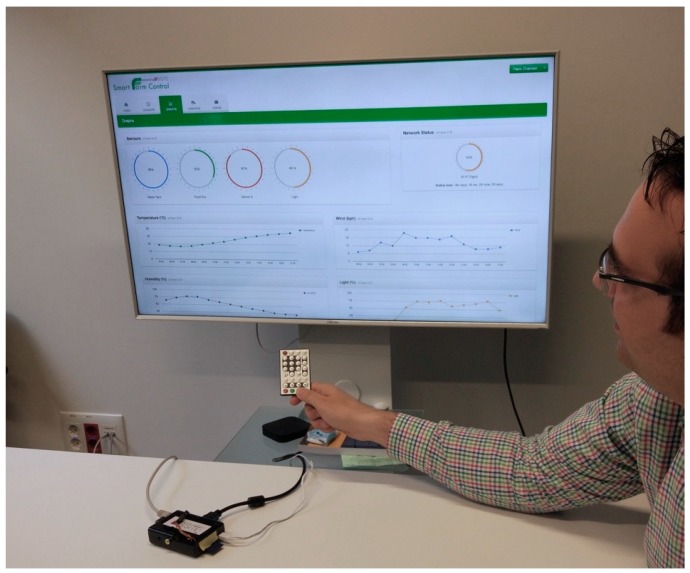
Cow average temperature, grouped by breeding gender.

The purpose of the farm remote monitoring is to allow the final users who participate in the case study to remotely view the data in an application. Most of the owners of agricultural holdings in the geographical area where the case study has been performed (La Fuente de San Esteban, Salamanca (40.8013959, −6.2624964)) are elderly people. Adults who live in rural zones are normally reluctant to the use of technology (and more specifically computers) as a central element to access data remotely. For this reason, it was proposed that the monitoring could be carried out from a television. Televisions are present in almost every household and can be operated in a simple way. This idea was provided by the local group *Asociación de Mayores San Esteban*. In this case of study, six individuals with ages between 40 and 64 years were offered for the development and validation of the monitoring prototype making use of TVs. In the development of the TV application, a Raspberry Pi with an IR sensor (model TSOP38238) has been used as hardware. The connection between the Raspberry Pi and the IR sensor is made using the GPIO pins, as shown in Figure 14.

**Figure 15:**
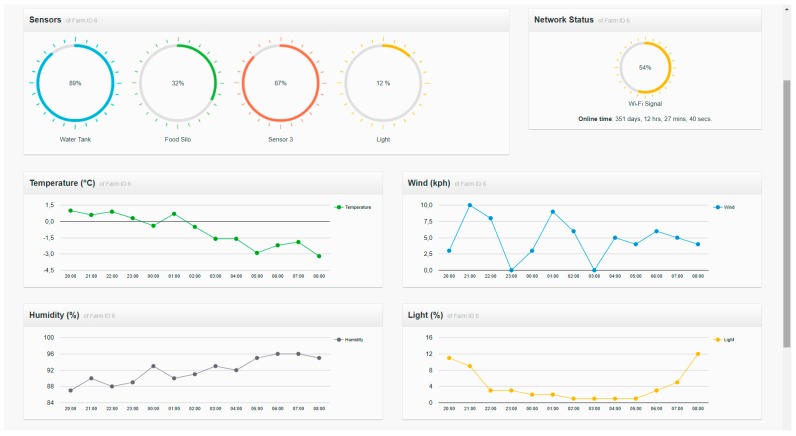
Television application interface.

Pulsations made in the control are used to operate the user’s television. This control interface allows users to access through the different options of the designed application. Users highlight the ease of use when accessing the data, since it is not necessary to install or operate a computer, simply plug in the TV and use the conventional control by pressing buttons. The software component designed to identify the buttons that act as an interface between the software and the hardware is based on LIRC (Linux Infrared Remote Control). It allows to associate the pressing of each button with a software event. The different buttons allow to view the different displays provided by the application as well as to move cameras that provide real-time images of the animals. Figure 15 shows the graphical user interface of the TV application. Users who tested and validated the TV prototype highlighted the simplicity of using it, as well as not having to do an initial investment in a computer.

**Figure 16:**
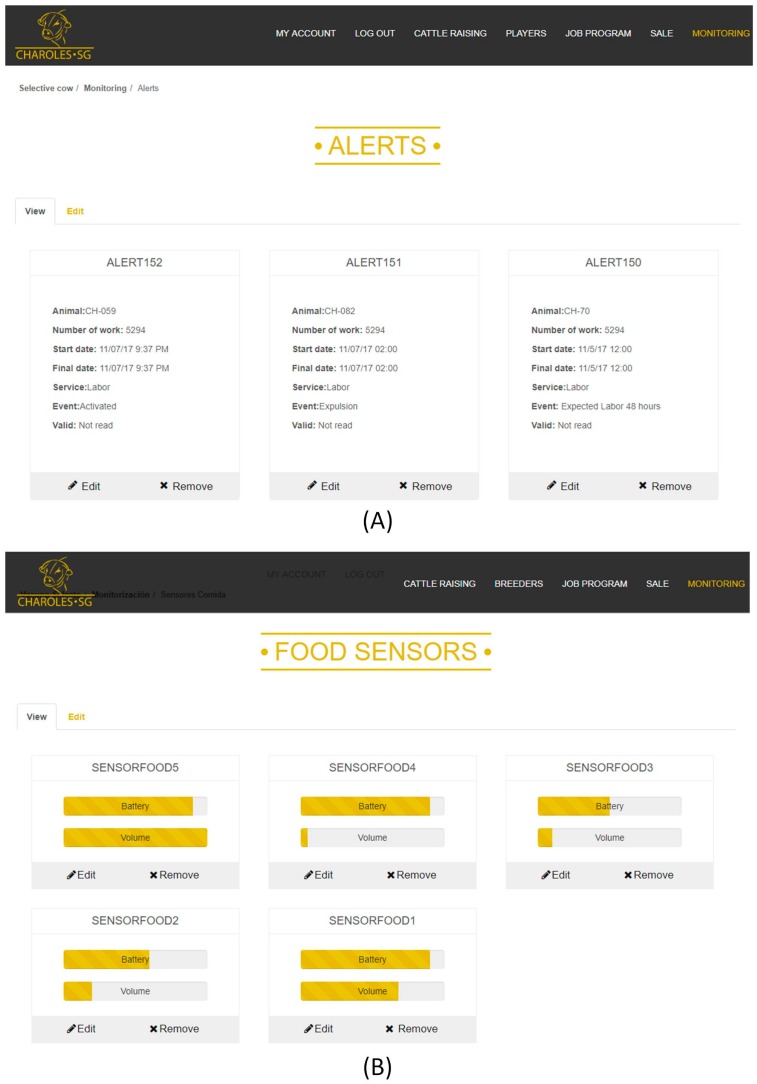
Web application interface.

The state of the livestock is also monitored through the web application. Figure 16A shows the information about the alerts that arrive to the system. In this image, you can see three alerts: the first of them indicates that the labor sensor has been activated (has begun to collect measures), the second indicates that there will be a labor in approximately 48 h and the third warns of the moment the labor begins. Figure 16B shows information about the food sensors, which includes the feed levels and the battery level of the sensor (although the devices are self-sufficient thanks to the use of solar panels that charge the batteries). In addition to the monitoring information, the website includes other information on livestock such as prizes, breeders, sales or cattle raising.

**Figure 17:**
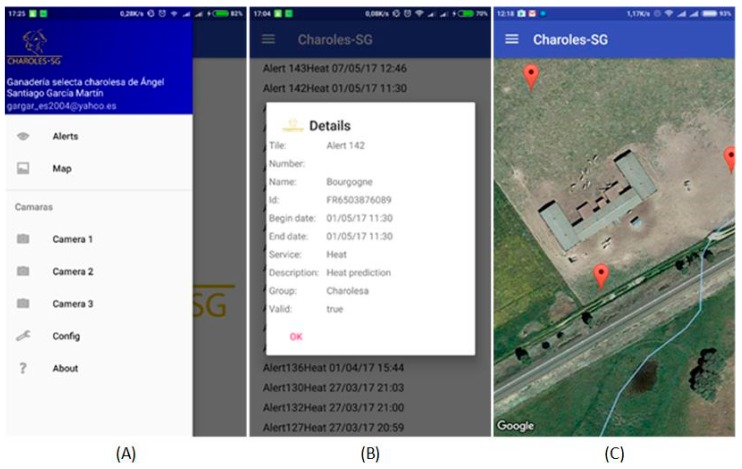
(**A**) The application menu; (**B**) a heat alert for a particular animal, in which you can see the animal’s name, id, the date and a description; and (**C**) the location of the animals.

The mobile application provides access to the most important functionality such as alerts on estrus, labor and feeding. The mobile application also allows getting notifications every time a new alert arrives to the system, thus it is not necessary to be continuously looking at the state of livestock. Notifications arrive on the mobile device regardless of whether the application is open or not. They are sent by the agents from the prediction organization. Through the mobile application, you can see alert information in real time, the location of the animals and the cameras. Figure 17A shows the application menu, Figure 17B shows a heat alert for a particular animal; the animal’s name, id, date and description can be read in the alert. Figure 17C shows the location of the animals. This location is loaded on a map with the satellite view of google maps, the location of the animals is redrawn synchronously by using websockets, reducing the traffic and updating the location only when there are data. In addition, the different chambers located on the farm can also be viewed from the application.

Cattle farmers have used the system for 12 months, emphasizing the simplicity and the enormous utility of the platform. They were happy with the possibility of monitoring the farm from their television, since the commercial systems that they knew, were based on the sending of SMS to their mobiles, which charge money. From the different utilities offered by the system, the farmers highlighted the benefit of the labor detection system since it allowed them to go to the field and assist the cows whenever it was necessary. The farmer felt overwhelmed by the number of data viewed on the TV application and it had to be reduced to make its use easier. The system for the detection of feeding anomalies, provided greater comfort, since the farmer did not have to spend that much time observing the cattle. The possibility of knowing the state of the feeders and the position of the cows avoids unnecessary displacements. The GPS position of the animal allows to find the animal quickly, which is very important in extensive farming where the farmer must search the animal with a vehicle and would normally spend a lot of time at the task.

## Figures and Tables

**Table 1:** Cattle Wearables.

Name	Type of Device	Utility	Technologies	Reference
Collar Silent Herdsman	Collar	Rumination, intake, fertility, temperature	-	[36]
AfiMilk MPC	Milk meter	Amount of production, milking trends, conductivity measurement	-	[37]
AfiTag Pedometer	Pedometer	Identification of animals, activity measurement, estrus detection	-	[38]
KV GPS system	Collar	Location of animals, (battery charge with photovoltaic system)	GPS	[39]
Allflex Tags	Ear tag	Identification of animals	HDX	[40]
Moocall Calving Sensor	Tail sensor	Prediction of births	GSM	[41]
Telenax Implant	Rumen implant	Theft detection	RF	[42]

**Table 2:** Relationship between the sensors and the analysis systems.

Sensor	Data Collected	Analysis/Prediction System Where It Is Used
GPS Sensor	Cattle Geolocation	System for the monitoring of ingestion and system for calving detection
Motion Sensor	Physical acceleration	System for the monitoring of ingestion and system for estrus detection
Vaginal thermometer	Corporal temperature	System for calving detection
Food sensor	Distance	System for the detection of food levels in the feeders

**Table 3:** Cows body temperature prior to delivery.

	Hours Before and After Calving (Hour 0 = Time of Calving)																			
	−144	−136	−128	−120	−112	−104	−96	−88	−80	−72	−64	−56	−48	−40	−32	−24	−16	−8	0	
Cow 1 Male	39.99	40.00	39.92	40.16	39.94	39.99	40.06	39.97	40.08	40.03	40.01	39.98	40.02	39.80	39.64	39.65	39.30	39.98	-	Cow Body C° Temperature
Cow 2 Female	39.99	39.58	39.8	39.82	39.85	40.01	39.83	39.57	39.83	39.84	40.03	39.83	40.05	39.83	39.62	39.62	39.32	39.83	-	
Cow 3 Male	40.10	40.20	40.18	40.06	40.30	40.60	40.02	39.85	40.52	40.57	40.14	40.85	40.47	39.82	39.81	39.38	39.39	39.89	-	
Cow 4 Female	39.29	39.3	39.79	39.17	39.05	39.3	39.04	39.69	39.48	39.69	39.3	39.91	39.88	39.78	38.86	39.17	38.33	39.47	-	
Cow 5 Male	40.23	40.23	40.24	40.09	40.30	40.43	40.78	40.00	40.10	40.46	40.52	40.48	40.48	40.72	40.16	40.20	39.34	40.10	-	
Cow 6 Female	40.31	40.2	40.26	40.68	39.93	40.01	40.58	40.1	40.33	40.33	40.11	40.09	40.67	40.1	40.11	39.2	39.88	39.96	-	

**Figure A1:**
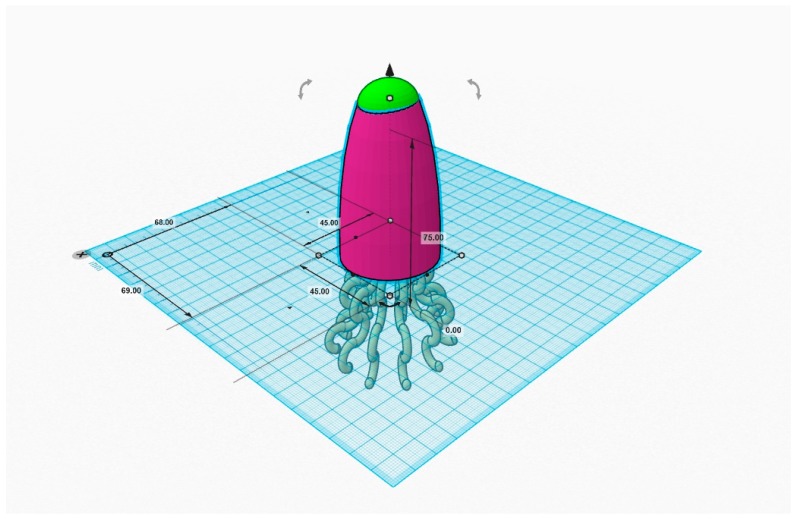
Design of the Vaginal Thermometer.

**Figure A2:**
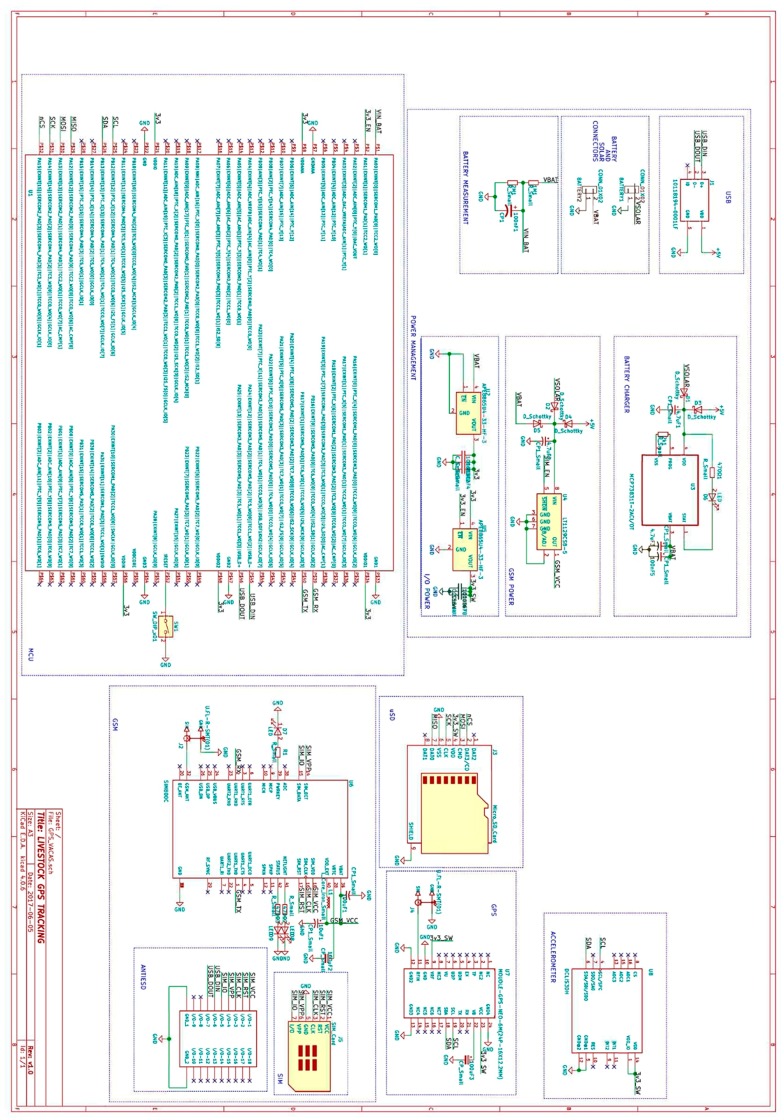
Electric Collar Scheme.

## Appendix A

Since other researchers may decide to use the hardware for their own experimentation, we decided to add electrical diagrams and the interconnection of the components designed for this case study, to this document.

### Appendix A.1. Design of the Vaginal Thermometer

Figure A1 shows the 3D design of the vaginal thermometer used in the system for calving detection.

### Appendix A.2. Electric Collar Scheme (GPS + Accelerometer)

Figure A2 shows the electric scheme of the collar that includes both GPS and motion sensors, used in the monitoring of ingestion, estrus detection and calving detection systems.
